# Global post-marketing safety surveillance of Tumor Treating Fields (TTFields) in patients with high-grade glioma in clinical practice

**DOI:** 10.1007/s11060-020-03540-6

**Published:** 2020-06-13

**Authors:** Wenyin Shi, Deborah T. Blumenthal, Nancy Ann Oberheim Bush, Sied Kebir, Rimas V. Lukas, Yoshihiro Muragaki, Jay-Jiguang Zhu, Martin Glas

**Affiliations:** 1 Department of Radiation Oncology, Thomas Jefferson University, Philadelphia, PA 19107 USA; 2 Neuro-Oncology Unit, Division of Oncology, Tel Aviv Sourasky Medical Center and Sackler School of Medicine, Tel Aviv University, Tel Aviv, Israel; 3 Department of Neurological Surgery and Neurology, University of California San Francisco, San Francisco, CA USA; 4 Division of Clinical Neurooncology, Department of Neurology, University Hospital Essen, University Duisburg-Essen, Essen, Germany; 5 West German Cancer Center, German Cancer Consortium, Partner Site Essen, University Hospital Essen, University Duisburg-Essen, Essen, Germany; 6 Department of Neurology, Northwestern University, Chicago, IL USA; 7 Lou & Jean Malnati Brain Tumor Institute at the Lurie Comprehensive Cancer Center, Northwestern University, Chicago, USA; 8 Faculty of Advanced Techno-Surgery, Institute of Advanced Biomedical Engineering and Science, Tokyo Women’s Medical University, Tokyo, Japan; 9 Department of Neurosurgery, Tokyo Women’s Medical University, Tokyo, Japan; 10 McGovern Medical School and Memorial Hermann Hospital at Texas Medical Center, University of Texas Health Science Center at Houston, Houston, TX USA

**Keywords:** TTFields, Glioblastoma, Real-world, Safety surveillance, Tolerability, Skin adverse events

## Abstract

**Introduction:**

Tumor Treating Fields (TTFields; antimitotic treatment) delivers low-intensity, intermediate-frequency, alternating electric fields through skin-applied transducer arrays. TTFields (200 kHz) was FDA-approved in glioblastoma (GBM), based on the phase 3 EF-11 (recurrent GBM, rGBM) and EF-14 (newly diagnosed GBM, ndGBM) trials. The most common TTFields-related adverse event (AE) in both trials was array-associated skin irritation. We now report on TTFields-related AEs in the real-world, clinical practice setting.

**Methods:**

Unsolicited, post-marketing surveillance data from TTFields-treated patients (October 2011–February 2019) were retrospectively analyzed using MedDRA v21.1 preferred terms, stratified by region (US, EMEA [Europe, Middle East, Africa], Japan), diagnosis (ndGBM, rGBM, anaplastic astrocytoma/oligodendroglioma, other brain tumors), and age (< 18 [pediatric], 18–64 [adults], ≥ 65 [elderly]; years of age).

**Results:**

Of 11,029 patients, 53% were diagnosed with ndGBM and 39% were diagnosed with rGBM at any line of disease recurrence. Most were adults (73%), 26% were elderly, and the male-to-female ratio was ~ 2:1 (close to published ratios of typical GBM populations). The most commonly reported TTFields-related AE was array-associated skin reaction, occurring in patients with ndGBM (38%), rGBM (29%), anaplastic astrocytoma/oligodendroglioma (38%), and other brain tumors (31%); as well as 37% of pediatric, 34% of adult, and 36% of elderly patients. Most skin AEs were mild/moderate and manageable. Other TTFields-related AEs in patients with ndGBM/rGBM included under-array heat sensation (warmth; 11%, 10%, respectively) and electric sensation (tingling; 11%, 9%, respectively), and headache (7%, 6%, respectively).

**Conclusions:**

This TTFields safety surveillance analysis in > 11,000 patients revealed no new safety concerns, with a favorable safety profile comparable with published TTFields/GBM trials. The safety profile remained consistent among subgroups, suggesting feasibility in multiple populations, including elderly patients.

**Graphic abstract:**

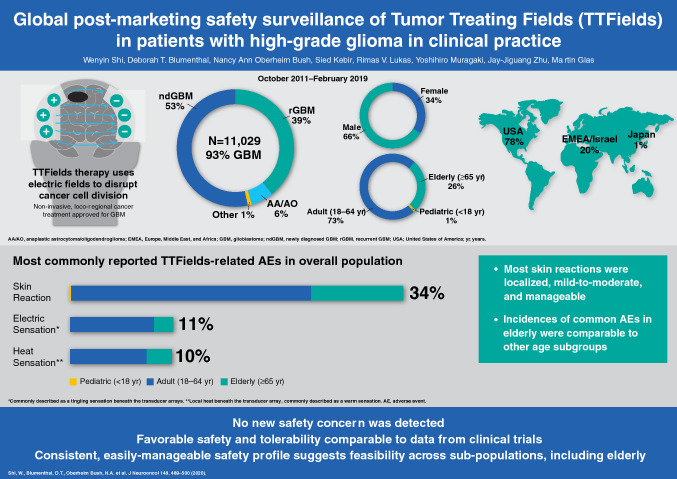

## Introduction

The most common primary brain/central nervous system (CNS) cancers in adults are malignant gliomas, including glioblastoma (GBM), anaplastic astrocytoma, and anaplastic oligodendroglioma [1]. GBM is an aggressive glioma that accounts for 15% of all primary brain/CNS tumors, 48% of primary malignant CNS tumors, and 57% of all gliomas in the United States (US) [2]. Although GBM is a rare tumor type with an estimated global GBM incidence of < 10 per 100,000, it has high mortality and an extremely poor prognosis [3, 4]. Moreover, GBM incidence is generally higher in men, with a male-to-female ratio ranging from 1.0 to 1.9 [2, 5]. In 2020, 13,140 new cases of GBM are projected in the US, including 6950 cases among people ≥ 65 years of age [2].

Prior to Tumor Treating Fields (TTFields), the first-line, standard of care (SOC) treatment for patients with newly diagnosed GBM (ndGBM) consisted of maximal safe resection followed by adjuvant radiotherapy (RT) plus concomitant and maintenance temozolomide (TMZ) chemotherapy [6–8]. Median overall survival (OS) for GBM ranges from 6.0 to 19.6 months, despite treatment advances in neurosurgery, RT, and chemotherapy [9]. The 1-year survival rate is 41%, ranging from 72% in patients who are 20–44 years of age to 31% in patients who are 65–74 years of age [2]. Based on positive phase 3 results, the new SOC treatment algorithm for ndGBM includes the addition of TTFields to maintenance TMZ [8, 10, 11]. In general, TTFields are a novel, noninvasive, antineoplastic therapy involving localized delivery of low-intensity (1–3 V/cm), intermediate-frequency (100–500 kHz), alternating electric fields that affect rapidly dividing cancer cells [12–15]. The fields are delivered continuously to the locoregional tumor bed using 2 pairs of skin-affixed transducer arrays.

Based on the phase 3 EF-11 and EF-14 clinical trial data, the US Food and Drug Administration (FDA) approved TTFields (200 kHz optimal frequency for GBM) for adults (≥ 22 years of age) as monotherapy for recurrent GBM (rGBM; 2011) and in combination with adjuvant post-chemoradiation TMZ for ndGBM (2015). In addition to US approval, global ndGBM and rGBM approvals for TTFields in adults (≥ 18 years of age) include Conformitè Europëenne (CE) Mark IIB in various Europe, Middle East, and Africa (EMEA) regions, the Pharmaceuticals and Medical Devices Agency (PMDA) Class III in Japan, and most recently (2020) by the National Medical Products Administration (NMPA) in China.

Notably, TTFields is classified as a category 1 recommendation in the National Comprehensive Cancer Network® guidelines for ndGBM [10] and is recognized as a treatment advancement in clinical cancer care by the American Society of Clinical Oncology [16]. Although the scope of guidance by the American Society of Radiation Oncology (2016) is on evidence-based radiotherapy or concomitant treatments to radiotherapy, TTFields was mentioned albeit limited published trial data at time of clinical evidence assessments for guideline inclusions (i.e., implemention of a priori exclusion of studies available only as abstracts). A planned, global clinical trial (TRIDENT) will assess the role of TTFields as an upfront treatment in combination with radiochemotherapy. European guidelines which incorporate TTFields include Spain’s Spanish Society of Medical Oncology (2017), England’s National Institute for Health and Care Excellence (2018), as well as the Swedish national guidelines for tumors in the brain and spinal cord (2020) that specifically endorses the addition of TTFields for GBM. European Society of Medical Oncology clinical practice guideline (2014) mentions TTFields, although the guideline is currently outdated, as it predates current clinical evidence available for TTFields. Moreover, the Society of Neuro-oncology and European Society of Neuro-Oncology joint consensus (2020) has described TTFields as a treatment that improves survival in ndGBM. Other global guidelines include China’s Glioma Treatment Guidelines (2018), which recommends TTFields for patients with GBM based on Level 1 evidence.

TTFields demonstrated efficacy in patients with ndGBM in the phase 3 EF-14 clinical trial [11]. Median progression-free survival was significantly improved to 6.7 months with TTFields plus TMZ versus 4.0 months with TMZ alone (hazard ratio [HR], 0.63; 95% confidence interval [CI], 0.52–0.76; p < 0.001). Median OS was significantly improved to 20.9 months versus 16.0 months from randomization (or from 24.5 months versus 19.8 months from diagnosis), respectively (HR, 0.63; 95% CI, 0.53–0.76; p < 0.001) [11]. The 5-year survival rate was 13% versus 5%, respectively (p = 0.004), a 2.6-fold increase [11]. In post hoc analyses of the EF-14 clinical trial, TTFields plus TMZ in all patient subgroups was associated with increased PFS and OS (Cox proportional hazards; p < 0.05 for the treatment effect within each subgroup), regardless of age, sex, Karnofsky performance score (KPS), O6-methylguanine-DNA methyltransferase promoter methylation status, geographic region, or extent of resection [11].

GBM almost universally recurs. Prior to TTFields, phase 2 and 3, rGBM clinical trials reported median OS after recurrence of only 6.0–9.8 months [17–23]. However, a post-hoc EF-14 analysis found that TTFields plus TMZ after first recurrence could prolong median OS versus TMZ alone for patients that were treated with TTFields beyond first progression (11.8 vs 9.2 months, respectively; HR, 0.70; 95% CI, 0.48–1.00; p = 0.049) [24].

TTFields was well-tolerated by patients in phase 3 trials; the most common treatment-related adverse event (AE) was localized skin AEs beneath the arrays [11, 21]. In the EF-14 trial, the majority of skin AEs (52%) were grade 1 or 2 (mild–to-moderate) and only 2% were grade 3 (severe) [11]. Systemic AE rates were similar with TTFields plus TMZ versus TMZ alone (48% vs 44%, respectively) and were consistent with previously reported TMZ clinical trials [7, 11, 25–27].

The most common AE in the Patient Registry Dataset (PRiDe), a post-marketing registry of 457 patients with rGBM who received TTFields in the US (October 2011–November 2013), was also array-associated mild-to-moderate skin irritation [28]. No new TTFields-related safety signals or systemic AEs were noted [28]. Overall, TTFields-related skin AEs based on registry were easily managed and treated with topical corticosteroids or antibiotics [21, 28] and typically did not require treatment interruption [21, 28].

This retrospective, unsolicited, global, post-market surveillance study aims to expand the real-world safety evidence for TTFields by analyzing AE profiles from a cohort of > 11,000 patients who received TTFields in the real-world, clinical practice settings within the US, EMEA, and Japan.

## Methods

Safety data were retrospectively collected from routine, post-marketing surveillance of patients with brain tumors treated with TTFields (October 2011–February 2019). AE data were not actively solicited, but collated from published literature screening and patient, caregiver, and/or prescriber reports during routine interactions with the device manufacturer (Novocure®; e.g., Device Support Specialist visits, prescriber interactions, and patient emails to nCompass™ support team). Subsequently, AEs were assessed by the Medical Safety Department as mandated by health authorities. AE reporting was based on the Medical Dictionary for Regulatory Activities version 21.1 (MedDRA v21.1) body system organ classes and preferred terms. Data were collected from patients treated with TTFields in the US, EMEA, and Japan. A subset of this data was previously published in the PRiDe registry. Safety data was provided by device manufacturer for current safety analyses. Since data were retrospectively gathered, AE severity could only be classified as nonserious or serious. An AE was considered serious if it led to ≥ 1 of the following: (1) death; (2) life-threatening illness/injury; (3) permanent body structure/function impairment; (4) in-patient hospitalization or prolongation of existing hospitalization; (5) medical/surgical intervention to prevent life-threatening illness, injury, or permanent body structure/function impairment; (6) fetal distress/death, congenital abnormality, or birth defect.

### Patients and treatment

Patients were 3–89 years of age and included those with brain tumors treated with TTFields between October 2011 and February 2019 in the US, EMEA, and Japan. Information on TTFields in combination with other cancer/concomitant treatments was not included, as data were collated from routine, retrospective, post-marketing surveillance reports, and combination data were not available for all patients. Hence, findings derived from these safety data cannot be deemed conclusive due to their observational nature. However, due to the large sample size, analyses were conducted to screen for new safety signals or trends relating to TTFields in the real-world clinical setting. Therefore, some patients may have received TTFields in combination with chemotherapy, immunotherapy, anti-vascular endothelial growth factor agents, and/or other concomitant treatments. It is important to note that safety effects of other treatments could not be adjusted for and should be considered when evaluating these data.

### Analyses

Data from the overall patient cohort were stratified by: (1) diagnosis, including ndGBM, rGBM, as well as off-label use for grade 3 glioma (anaplastic astrocytoma and anaplastic oligodendroglioma) and other brain tumors (including brain metastases); (2) region (US, EMEA, and Japan); and (3) age group (3 to 17 years of age, pediatric [off-label use]; 18 to 64 years of age [in the US, off-label for ages 18–21], adult; and 65 to 89 years of age, elderly). Novocure’s Medical Safety department assessed AE relatedness to treatment; incidence was based on the number of unique patients reporting an AE. Moreover, due to the retrospective study design, additional types of patient information could not be analyzed. This was largely due to the non-prespecified nature of innate study design, in which patient AE reports were unsolicited. Also, routine, global post-marketing surveillance activities have no uniform mandate, as they often lack additionally collected patient information and separate documentation.

## Results

### Baseline characteristics

**Fig. 1:**
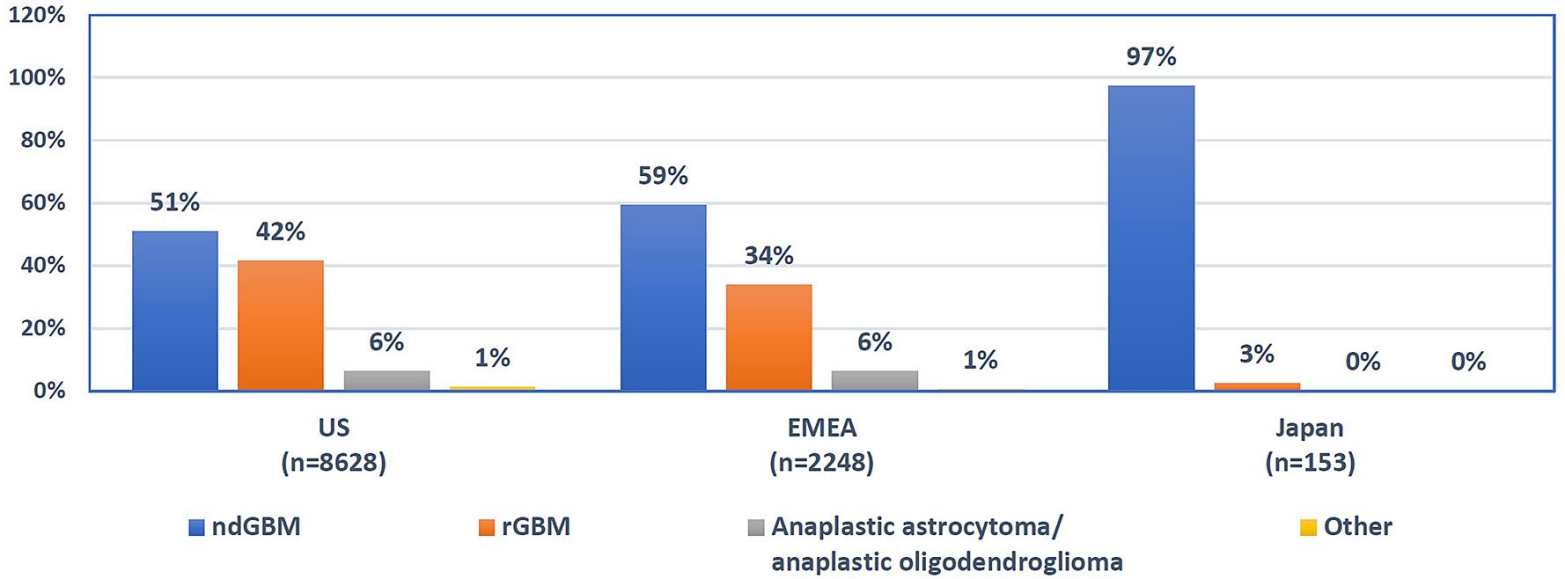
Proportion of patients who received TTFields by diagnosis and region. *EMEA* Europe, the Middle East, and Africa, *ndGBM* newly diagnosed glioblastoma, *rGBM* recurrent glioblastoma, *TTFields* Tumor Treating Fields, *US* United States. ^a^Other brain tumors, including brain metastases from different cancer types

Safety reports were received during post-marketing surveillance of 11,029 patients treated with TTFields. The majority of TTFields-treated patients were diagnosed with ndGBM (53%) or rGBM (39%) (Table 1). Median age at TTFields initiation was 57 years of age (range: 3–89). Approximately three-quarters (73.4%) of patients were adults (≥ 18 to < 65 years of age), and 26.2% were elderly (≥ 65 years of age; Table 1). Although TTFields are not currently approved for pediatric use, 52 (0.5%) pediatric patients were identified in the total cohort. Age stratification of total cohort showed that the most common diagnosis among pediatric patients was rGBM (42.3%), while the majority of adult (50.2%) and elderly (62.5%) patients were diagnosed with ndGBM (Table 1). Few patients (~ 7%) were treated off-label with TTFields for anaplastic astrocytoma and anaplastic oligodendroglioma or other brain tumors (including brain metastases from different cancer types). Regionally, 78.2% of patients were treated in the US, 20.4% in EMEA regions, and 1.4% in Japan. In all three regions, more patients with ndGBM than rGBM received treatment with TTFields (Fig. 1). The very high rate of ndGBM diagnoses in Japan (97.4%) is likely a result of Japan’s national government health insurance only covering reimbursement for ndGBM treatment, despite approval of TTFields for ndGBM and rGBM treatment by the Ministry of Health, Labour and Welfare in Japan. Overall, the 1.97:1 (~ 2:1) male-to-female patient ratio (66.3% [n = 7313] to 33.7% [n = 3716], respectively), although on the higher-end of range, was closely representative of published ratios of a typical GBM population [2, 5].

**Table 1:** Baseline characteristics of the TTFields-treated population (N = 11,029)

Characteristic	Total	ndGBM	rGBM	AA/AO	Other^a^
N/n (%)	N = 11,029 (100.0)	n = 5887 (53.4)	n = 4345 (39.4)	n = 682 (6.2)	n = 115 (1.0)
**Age (years), n (%)**					
< 18	52 (0.5)	19 (0.3)	22 (0.5)	8 (1.2)	3 (2.6)
18 to 64	8090 (73.4)	4063 (69.0)	3337 (76.8)	596 (87.4)	94 (81.7)
≥ 65	2887 (26.2)	1805 (30.7)	986 (22.7)	78 (11.4)	18 (15.7)
**Gender, n (%)**					
Male	7313 (66.3)	3849 (65.4)	2921 (67.2)	470 (68.9)	73 (63.5)
Female	3716 (33.7)	2038 (34.6)	1424 (32.8)	212 (31.1)	42 (36.5)
**Region, n (%)**					
United States	8628 (78.2)	4402 (74.8)	3583 (82.5)	540 (79.2)	103 (89.6)
EMEA	2248 (20.4)	1336 (22.7)	758 (17.4)	142 (20.8)	12 (10.4)
Japan	153 (1.4)	149 (2.5)	4 (0.1)	0	0

Note: all proportions do not equal 100%, due to rounding to the tenths decimal

*AA* anaplastic astrocytoma, *AO* anaplastic oligodendroglioma, *EMEA* Europe, the Middle East, and Africa, *ndGBM* newly diagnosed glioblastoma, *rGBM* recurrent glioblastoma, *TTFields* Tumor Treating Fields

^a^Other brain tumors including brain metastases from different cancer types

### Safety

Overall, 63% of patients treated with TTFields reported ≥ 1 AE (Table 2). Stratification by diagnosis showed similar proportions of patients with ndGBM (65%), rGBM (60%), anaplastic astrocytoma and anaplastic oligodendroglioma (65%), and other brain tumors (61%) reporting ≥ 1 AE. When stratified by age, 58% of pediatric, 63% of adult, and 66% of elderly patients reported ≥ 1 AE. In general, AEs with a reported incidence of ≥ 5% in the total cohort were comparable across diagnostic and age subgroups. Skin disorders (36%), general disorders/application site conditions (31%), and nervous system disorders (27%) were the most commonly reported AEs among all patients, occurring at analogous rates across diagnostic and age subgroups. General physical health deterioration rates were similarly low across diagnostic and age subgroups (0%–4%; data not shown).

**Table 2:** Most common AEs in TTFields-treated patients by diagnosis and age, with incidence of  ≥ 5% in the total cohort

MedDRA v21.1 system organ class/preferred term	Total	**Diagnosis**				**Age (years)**		
		ndGBM	rGBM	AA/AO	Other^c^	< 18	18 to 64	≥ 65
**N/n**	N = 11,029	n = 5887	n = 4345	n = 682	n = 115	n = 52	n = 8090	n = 2887
**Patients with ≥ 1 AE, n (%)**	6985 (63)	3847 (65)	2625 (60)	443 (65)	70 (61)	30 (58)	5061 (63)	1894 (66)
**General, n (%)**	3460 (31)	1940 (33)	1263 (29)	223 (33)	34 (30)	18 (35)	2561 (32)	881 (31)
Electric sensation^a^	1172 (11)	673 (11)	402 (9)	77 (11)	20 (17)	7 (13)	948 (12)	217 (8)
Fatigue/malaise	589 (5)	353 (6)	198 (5)	34 (5)	4 (3)	4 (8)	401 (5)	184 (6)
Heat sensation^b^	1153 (10)	619 (11)	439 (10)	86 (13)	9 (8)	9 (17)	862 (11)	282 (10)
Pain	670 (6)	413 (7)	212 (5)	41 (6)	4 (3)	5 (10)	475 (6)	190 (7)
**Injury, n (%)**	938 (9)	477 (8)	394 (9)	55 (8)	12 (10)	2 (4)	606 (7)	330 (11)
Fall	706 (6)	343 (6)	311 (7)	43 (6)	9 (8)	2 (4)	439 (5)	265 (9)
**Nervous system, n (%)**	2924 (27)	1571 (27)	1160 (27)	168 (25)	25 (22)	14 (27)	2145 (27)	765 (26)
Headache	859 (8)	467 (8)	324 (7)	60 (9)	8 (7)	4 (8)	668 (8)	187 (6)
Seizure	1203 (11)	631 (11)	495 (11)	68 (10)	9 (8)	7 (13)	891 (11)	305 (11)
**Skin, n (%)**	3985 (36)	2345 (40)	1334 (31)	270 (40)	36 (31)	19 (37)	2888 (36)	1078 (37)
Skin reaction	3805 (34)	2251 (38)	1258 (29)	260 (38)	36 (31)	19 (37)	2748 (34)	1038 (36)

*AA* anaplastic astrocytoma, *AE* adverse event, *AO* anaplastic oligodendroglioma, *ndGBM* newly diagnosed glioblastoma, *rGBM* recurrent glioblastoma, *TTFields* Tumor Treating Fields

^a^Commonly described as a tingling sensation beneath the transducer arrays

^b^Local heat beneath the transducer array, commonly described as a warm sensation

^c^Other brain tumors including brain metastases from different cancer types

Skin reactions beneath the transducer arrays were the most commonly reported TTFields-related AEs (34%) among the total cohort population (Table 2). The incidence of treatment-related skin reactions was greater in patients with ndGBM versus rGBM (38% vs 29%, respectively), yet rates were consistent for pediatric, adult, and elderly age subgroups (37%, 34%, and 36%, respectively). Other AEs potentially related to TTFields included electric sensation (i.e., under-array tingling; 11%), heat sensation (i.e., under-array warmth; 10%), and headache (6%) (Table 2). Electric sensation may be attributable to displaced contact of the transducers arrays on the scalp, which perhaps could result in arching across the gap between the arrays and scalp interface (Table 3). Reports of electric sensations ranged from 8%–13% across age subgroups. Overall, elderly patients, who are ~ 1/3 of the total sample size that includes pediatric and adult patients, reported the lowest incidences of electric sensations, heat sensations, and headaches across age subgroups (Table 3). Observed lower incidences of electric and heat sensations in elderly are perhaps attributable to less robust hair growth and decreased sweating. Electric sensation was also reported by similar proportions of patients diagnosed with ndGBM and rGBM (11% and 9%, respectively). Heat sensations were reported in 17% of pediatric and 10% of elderly patients. Headaches were infrequent across all subgroups and can also be a result of or attributable to underlying brain cancer disease symptomology (4–8%; Table 3).

**Table 3:** Most common TTFields-related AEs with an incidence of ≥ 2% in total patient cohort, by diagnosis and age

MedDRA v21.1 preferred term	Total	**Diagnosis**				**Age (years)**		
		ndGBM	rGBM	AA/AO	Other^c^	< 18	18 to 64	≥ 65
N/n	N = 11,029	n = 5887	n = 4345	n = 682	n = 115	n = 52	n = 8090	n = 2887
Patients with ≥ 1 AE, n (%)	5260 (48)	3022 (51)	1835 (42)	347 (51)	56 (49)	27 (52)	3842 (47)	1391 (48)
Skin reaction	3805 (34)	2251 (38)	1258 (29)	260 (38)	36 (31)	19 (37)	2748 (34)	1038 (36)
Electric sensation^a^	1169 (11)	673 (11)	399 (9)	77 (11)	20 (17)	7 (13)	945 (12)	217 (8)
Heat sensation^b^	1153 (10)	619 (11)	439 (10)	86 (13)	9 (8)	9 (17)	862 (11)	282 (10)
Headache	716 (6)	396 (7)	264 (6)	51 (7)	5 (4)	4 (8)	559 (7)	153 (5)
Pain	484 (4)	304 (5)	149 (3)	28 (4)	3 (3)	2 (4)	349 (4)	133 (5)
Fatigue/malaise	395 (4)	248 (4)	115 (3)	29 (4)	3 (3)	3 (6)	260 (3)	132 (5)
Discomfort	201 (2)	116 (2)	70 (2)	11 (2)	4 (3)	3 (6)	145 (2)	53 (2)

*AA* anaplastic astrocytoma, *AE* adverse event, *AO* oligodendroglioma, *MedDRA* Medical Dictionary for Regulatory Activities, *ndGBM* newly diagnosed glioblastoma, *rGBM* recurrent glioblastoma, *TTField*s Tumor Treating Fields

^a^Commonly described as a tingling sensation beneath the transducer arrays

^b^Local heat beneath the transducer array, commonly described as a warm sensation

^c^Other brain tumors including brain metastases from different cancer types

Other device-related skin AEs included hyperhidrosis, wound complication (including dehiscence and infection), hypersensitivity, and skin erosions/ulcers (each ≤ 1%) (Table 4). Moreover, only 27 unique patients (< 1%) experienced serious device-related skin AEs (Table 5). Serious skin AEs included wound complication, skin erosion, skin ulcer, and skin laceration. Rates of balance disorders, falls, and fractures that were deemed related to TTFields device usage were low and comparable for all diagnostic and age subgroups (each ≤ 1%). To assess the potential impact of device-related falls on increased age-related incidences, a breakdown of number of falls by age distribution in decades (years of age; 11–20, 21–30, 31–40, 41–50, 51–60, 61–70, 71–80, and 81–90) was calculated. The percent ratios of patients with TTFields device-related falls to total falls by age distribution in decades were 0%, 11%, 0%, 6%, 9%, 9%, 4%, and 8%, respectively.

**Table 4:** TTFields-related AEs, by diagnosis and age (full data set)

MedDRA v21.1 system organ class/preferred term	Total	**Diagnosis**				**Age (years)**		
		ndGBM	rGBM	AA/AO	Other^c^	< 18	18 to 64	≥ 65
**N/n**	N = 11,029	n = 5887	n = 4345	n = 682	n = 115	n = 52	n = 8090	n = 2887
**Patients with ≥ 1 AE, n (%)**	5260 (48)	3022 (51)	1835 (42)	347 (51)	56 (49)	27 (52)	3842 (47)	1391 (48)
**Ear, n (%)**	3 (< 1)	2 (< 1)	–	1 (< 1)	–	–	1 (< 1)	2 (< 1)
Auditory disorder	3 (< 1)	2 (< 1)	–	1 (< 1)	–	–	1 (< 1)	2 (< 1)
**Eye, n (%)**	3 (< 1)	3 (< 1)	–	–	–	–	2 (< 1)	1 (< 1)
Eye disorder	3 (< 1)	3 (< 1)	–	–	–	–	2 (< 1)	1 (< 1)
**Gastrointestinal, n (%)**	6 (< 1)	3 (< 1)	2 (< 1)	–	1 (1)	–	6 (< 1)	–
Nausea/vomiting	6 (< 1)	3 (< 1)	2 (< 1)	–	1 (1)	–	6 (< 1)	–
**General, n (%)**	2683 (24)	1533 (26)	936 (22)	183 (27)	31 (27)	16 (31)	2013 (25)	654 (23)
Chills	1 (< 1)	–	–	1 (< 1)	–	–	–	1 (< 1)
Complication associated with device	4 (< 1)	3 (< 1)	1 (< 1)	–	–	–	1 (< 1)	3 (< 1)
Discomfort	201 (2)	116 (2)	70 (2)	11 (2)	4 (3)	3 (6)	145 (2)	53 (2)
Electric sensation^a^	1169 (11)	673 (11)	399 (9)	77 (11)	20 (17)	7 (13)	945 (12)	217 (8)
Fatigue/malaise	395 (4)	248 (4)	115 (3)	29 (4)	3 (3)	3 (6)	260 (3)	132 (5)
Gait disturbance	8 (< 1)	4 (< 1)	4 (< 1)	–	–	–	7 (< 1)	1 (< 1)
Heat sensation^b^	1153 (10)	619 (11)	439 (10)	86 (13)	9 (8)	9 (17)	862 (11)	282 (10)
Edema	4 (< 1)	4 (< 1)	–	–	–	–	2 (< 1)	2 (< 1)
Edema peripheral	8 (< 1)	8 (< 1)	–	–	–	–	7 (< 1)	1 (< 1)
Pain	484 (4)	304 (5)	149 (3)	28 (4)	3 (3)	2 (4)	349 (4)	133 (5)
Pyrexia	1 (< 1)	–	1 (< 1)	–	–	-	1 (< 1)	–
QoL decreased	77 (1)	61 (1)	14 (< 1)	2 (< 1)	–	–	58 (1)	19 (1)
**Immune system, n (%)**	16 (< 1)	15 (< 1)	–	1 (< 1)	–	–	13 (< 1)	3 (< 1)
Hypersensitivity	16 (< 1)	15 (< 1)	–	1 (< 1)	–	–	13 (< 1)	3 (< 1)
**Infections, n (%)**	11 (< 1)	9 (< 1)	1 (< 1)	1 (< 1)	–	–	8 (< 1)	3 (< 1)
Abscess	2 (< 1)	1 (< 1)	1 (< 1)	–	–	–	2 (< 1)	–
Infection	9 (< 1)	8 (< 1)	–	1 (< 1)	–	–	6 (< 1)	3 (< 1)
**Injury, n (%)**	156 (1)	92 (2)	53 (1)	9 (1)	2 (2)	–	103 (1)	53 (2)
Contusion	18 (< 1)	13 (< 1)	5 (< 1)	–	–	–	14 (< 1)	4 (< 1)
Fall	52 (< 1)	22 (< 1)	24 (1)	6 (1)	–	–	34 (< 1)	18 (1)
Fracture	3 (< 1)	3 (< 1)	–	–	–	–	3 (< 1)	–
Injury	9 (< 1)	6 (< 1)	3 (< 1)	–	–	–	5 (< 1)	4 (< 1)
Muscle strain	1 (< 1)	1 (< 1)	–	–	–	–	1 (< 1)	–
Radiation injury	1 (< 1)	1 (< 1)	–	–	–	–	1 (< 1)	–
Skin abrasion	2 (< 1)	1 (< 1)	1 (< 1)	–	–	–	–	2 (< 1)
Skin laceration	42 (< 1)	27 (< 1)	13 (< 1)	2 (< 1)	–	–	27 (< 1)	15 (< 1)
Wound complication	46 (< 1)	29 (< 1)	13 (< 1)	2 (< 1)	2 (2)	–	33 (< 1)	13 (< 1)
**Musculoskeletal, n (%)**	37 (< 1)	15 (< 1)	19 (< 1)	3 (< 1)	–	–	30 (< 1)	7 (< 1)
Muscle spasms	5 (< 1)	4 (< 1)	1 (< 1)	–	–	–	4 (< 1)	1 (< 1)
Muscle twitching	24 (< 1)	8 (< 1)	13 (< 1)	3 (< 1)	–	–	21 (< 1)	3 (< 1)
Muscular weakness	7 (< 1)	3 (< 1)	4 (< 1)	–	–	–	4 (< 1)	3 (< 1)
Musculoskeletal stiffness	1 (< 1)	–	1 (< 1)	–	–	–	1 (< 1)	–
**Nervous system, n (%)**	739 (7)	406 (7)	275 (6)	52 (8)	6 (5)	4 (8)	580 (7)	155 (5)
Balance disorder	7 (< 1)	5 (< 1)	2 (< 1)	–	–	–	7 (< 1)	–
Cognitive disorder	2 (< 1)	1 (< 1)	1 (< 1)	–	–	–	2 (< 1)	–
Coordination abnormal	1 (< 1)	–	1 (< 1)	–	–	–	1 (< 1)	–
Dizziness	3 (< 1)	3 (< 1)	–	–	–	–	3 (< 1)	–
Dysesthesia	14 (< 1)	6 (< 1)	6 (< 1)	1 (< 1)	1 (1)	–	12 (< 1)	2 (< 1)
Headache	716 (6)	396 (7)	264 (6)	51 (7)	5 (4)	4 (8)	559 (7)	153 (5)
Hyperesthesia	1 (< 1)	–	–	1 (< 1)	–	–	1 (< 1)	–
Paresthesia	3 (1)	3 (< 1)	–	–	–	–	3 (< 1)	–
Syncope	1 (< 1)	-	1 (< 1)	–	–	–	1 (< 1)	–
**Psychiatric, n (%)**	76 (1)	53 (1)	20 (< 1)	3 (< 1)	–	–	54 (1)	22 (1)
Agitation	7 (< 1)	3 (< 1)	4 (< 1)	–	–	–	4 (< 1)	3 (< 1)
Anxiety	7 (< 1)	4 (< 1)	3 (< 1)	–	–	–	4 (< 1)	3 (< 1)
Claustrophobia	3 (< 1)	2 (< 1)	–	1 (< 1)	–	–	3 (< 1)	–
Confusional state	1 (< 1)	1 (< 1)	–	–	–	–	1 (< 1)	–
Depression	8 (< 1)	6 (< 1)	2 (< 1)	–	–	–	7 (< 1)	1 (< 1)
Insomnia	50 (< 1)	39 (1)	9 (< 1)	2 (< 1)	–	–	35 (< 1)	15 (1)
Mental status change	1 (< 1)	–	1 (< 1)	–	–	–	1 (< 1)	–
Mood altered	1 (< 1)	1 (< 1)	–	–	–	–	1 (< 1)	–
Sleep disorder	1 (< 1)	–	1 (< 1)	–	–	–	1 (< 1)	–
Stress	3 (< 1)	2 (< 1)	1 (< 1)	–	–	–	2 (< 1)	1 (< 1)
**Skin, n (%)**	3895 (35)	2300 (39)	1295 (30)	264 (39)	36 (31)	19 (37)	2817 (35)	1059 (37)
Alopecia	3 (< 1)	3 (< 1)	–	–	–	–	3 (< 1)	–
Decubitus ulcer	2 (< 1)	1 (< 1)	–	1 (< 1)	–	1 (2)	–	1 (< 1)
Hyperhidrosis	144 (1)	90 (2)	45 (1)	9 (1)	–	–	115 (1)	29 (1)
Skin erosion	4 (< 1)	3 (< 1)	1 (< 1)	–	–	–	2 (< 1)	2 (< 1)
Skin reaction	3805 (34)	2251 (38)	1258 (29)	260 (38)	36 (31)	19 (37)	2748 (34)	1038 (36)
Skin ulcer	80 (1)	41 (1)	32 (1)	6 (1)	1 (1)	1 (2)	50 (1)	29 (1)
Hypersensitivity	16 (< 1)	15 (< 1)	–	1 (< 1)	–	–	13 (< 1)	3 (< 1)
**Vascular, n (%)**	2 (< 1)	1 (< 1)	1 (< 1)	–	–	–	–	2 (< 1)
Hematoma	1 (< 1)	1 (< 1)	–	–	–	–	–	1 (< 1)
Hemorrhage	1 (< 1)	–	1 (< 1)	–	–	–	–	1 (< 1)

*AA* anaplastic astrocytoma, *AE* adverse event, *AO* anaplastic oligodendroglioma, *MedDRA* Medical Dictionary for Regulatory Activities, *ndGBM* newly diagnosed glioblastoma, *QoL* quality-of-life, *rGB*M recurrent glioblastoma, *TTFields* Tumor Treating Fields

^a^Commonly described as a tingling sensation beneath the transducer arrays

^b^Local heat beneath the transducer array, commonly described as a warm sensation

^c^Other brain tumors including brain metastases from different cancer types

**Table 5:** Incidence of TTFields-related serious AEs in the total patient cohort

MedDRA v21.1 system organ class/preferred term	Total N = 11,029
**Patients with ≥ 1 reported AE, n (%)**	34 (< 1)
**Injury, n (%)**	24 (< 1)
Contusion	1 (< 1)
Fall	2 (< 1)
Fracture	2 (< 1)
Radiation injury	1 (< 1)
Skin laceration	2 (< 1)
Wound complication	19 (< 1)
**Skin, n (%)**	7 (< 1)
Skin erosion	2 (< 1)
Skin ulcer	5 (< 1)
**Nervous system, n (%)**	3 (< 1)
Headache	3 (< 1)
**Infections, n (%)**	1 (< 1)
Abscess	1 (< 1)

*AE* adverse event, *MedDRA* medical dictionary for regulatory activities, *TTFields* Tumor Treating Fields

Moreover, only 0–1% of patients across subgroups reported an AE of TTFields-related ‘quality of life (QoL) decreased’. It is important to note that ‘QoL decreased’, in the context of these analyses, refers to an unsolicited AE incidence of ‘QoL decreased’ per MedDRA v21.1 preferred terms and is not by any means a reference to any standardized, validated QoL assessment.

## Discussion

We retrospectively assessed the safety of TTFields in real-world, clinical practice settings, using global post-marketing surveillance data from a large patient cohort. Patient diagnoses included ndGBM, rGBM, and off-label treatment for anaplastic astrocytoma and anaplastic oligodendroglioma as well as metastatic brain tumors. Also, the male-to-female ratio of 1.97:1 was generally representative of global GBM populations, although slightly on the higher limit of the range. This slightly increased male predominance perhaps could be attributable to a number of factors, including sex hormones playing a relevant role, more women who do not want to shave their heads than men, or general population variability during the period that patients were retrospectively evaluated. It is important to note that the presumption that more women than men do not agree to shaving their head for aesthetic purposes, to noninvasively treat a very aggressive form of cancer, has not been evaluated to date and is speculative. Our results confirm the overall tolerability of TTFields, reveal no new safety signals, and demonstrate comparable safety and tolerability to that reported in the phase 3 TTFields/GBM clinical trials. As expected from our experiences with the EF-11 [21] and EF-14 [11] clinical trials and the PRiDe US post-marketing registry [28], mild-to-moderate localized skin AEs beneath the transducer arrays are the most commonly reported TTFields-associated AEs. Interestingly, there is no Common Terminology Criteria for AE (CTCAE) to date that is close to grading the severity of skin reactions, inclusive of irritant contact dermatitis. However, it is noteworthy to mention that a modified TTFields-specific skin reaction grading system is typically utilized to more accurately grade the severity of skin AEs, such as irritant contact dermatitis. Overall, it is comparable to the general AE severity grading guidelines. The modified grading system for TTFields-related skin reaction severity are divided into 4 categories. Grading categories are defined as: (1) Grade 1 (mild/asymptomatic symptoms)—no intervention required or only topical treatment intervention indicated; treatment interruption of < 3 days may be required; (2) Grade 2 (moderate symptoms)—systemic therapy required or event is requiring interruption of TTFields for > 3 days; (3) Grade 3 (severe/medically significant)—not immediately life threatening; hospitalization or prolongation of existing hospitalization indicated; and (4) Grade 4 (life threatening consequences)—urgent intervention indicated.

Skin AEs manifest as localized skin reactions beneath the transducer arrays. As previously reported in the literature, these skin AEs (irritant contact dermatitis) are most probably related to chronic skin exposure to irritants in transducer arrays, which are applied noninvasively to deliver TTFields through the skin to the tumor bed [29–31]. AE rates were comparable between adult and elderly patients and were lower in pediatric patients, likely due to the smaller pediatric sample size. The frequency of patients reporting ≥ 1 AE were similar across ndGBM, rGBM, and other gliomas, suggesting no observable differences between pathologic diagnosis and AE rates. Similar to results from clinical trials, no notable TTFields-related systemic AEs were detected. For example, in the EF-14 ndGBM trial, TTFields plus TMZ combination did not lead to increased systemic AEs compared with TMZ alone, suggesting that systemic toxicity was TMZ-related [11].

These data suggest tolerability and the feasibility of long-term TTFields treatment exposure to potentially improve treatment adherence and patient outcomes. As the overall TTFields dose is also related to usage and has been shown to correlate directly with OS, these data are significant for demonstrating that treatment adherence is critical for improved survival outcomes [32, 33]. In this context, it is worth noting that the effects of TTFields cease when the device system is powered off and that TTFields have no half-life, bioavailability, drug-drug interactions, or other pharmacokinetic parameters to consider.

In our analysis, AE incidence in general was lowest in pediatric patients (most likely associated with the smaller pediatric sample size). Observed AE incidences were comparable in the larger sample sizes of adult and elderly patients. In particular, incidence of skin reactions were similar for pediatric, adult, and elderly patients. TTFields-related AE rates of balance disorders, falls, and fractures across age groups were low (≤ 1% each; Table 4). These data also suggest that TTFields usage did not lead to excess AEs among elderly patients. Furthermore, the percent ratios of patients with TTFields device-related falls to total falls suggests no observable age-related patterns or incremental increases in device-related falls in geriatric subgroups relative to other age subgroups. Moreover, elderly patients showed the lowest observed incidence of both electric and heat sensations, headache, and discomfort across age subgroups. Based on these observations, KPS and Eastern Cooperative Oncology Group Performance Score (ECOG-PS), as well as the Lansky score for children and the Global Assessment of Functioning scores or other patient-related outcomes/factors may be more relevant and better scaled indicators for TTFields usage by patients with brain cancers in comparison to age. These performance status scales may be better means to quantify each cancer patient’s general well-being and daily-life activities to determine feasibility of TTFields treatment. TTFields treatment of elderly patient with ndGBM has been described as promising, since TTFields plus adjuvant chemotherapy compared with chemotherapy alone suggests improved OS in elderly patients with ndGBM. However, further evidence on QoL and tolerability in elderly patients is needed [34]. Our real-world evidence of evaluated safety/tolerability data for TTFields in elderly patients with GBM aids in defining unmet needs in this select population. Given these positive benefits and aforementioned increased disease incidence in the geriatric population, TTFields show potential as a feasible and favorable treatment modality for elderly patients with GBM.

TTFields-associated AEs are very different from chemotherapy-related AEs, a critical consideration in a patient population burdened with disease and symptomology, such as debilitating neurological symptoms and deficits elicited by the tumor itself [35] and unpleasant and sometimes life-threatening AEs related to radiation, chemotherapy, and other investigational treatments [36, 37]. To date, TTFields as a locoregional treatment modality do not induce systemic toxicity. Chemotherapy-treated patients commonly suffer from hematologic, gastrointestinal, and renal toxicity, as well as severe nausea, vomiting, headache, and fatigue [38]. In contrast, an EF-14 secondary analysis found that concomitant TTFields with TMZ in patients with ndGBM did not result in reduced health-related QoL (HRQoL) versus TMZ alone, except for increased incidence of itchy skin, which was expected [39]. This lack of negative influence on HRQoL further supports the utility of combining TTFields with other GBM treatments [39].

This study has a large cohort as its major strength, despite limitations based on its retrospective design. Analyses were not statistically powered based on study design and therefore comparative statements should be deemed observational in nature. For instance, AEs were reported as serious or nonserious with no AE grading system, since data were not collected from a clinical trial. TTFields should be administered continuously; however patient adherence to treatment was not specified and perhaps may have impacted occurrence of related AEs. In addition, we did not report on concurrent and concomitant therapies that may have likely inflated AE incidences; such as steroid usage, which could affect the fragility of skin in relation to TTFields application. Since safety data were collated only from TTFields-treated patients that reported AEs, incidence of the overall cohort and subgroups is likely overestimated. Finally, no efficacy, survival, or standardized QoL assessment data were included.

## Conclusions

This retrospective analysis of post-marketing surveillance data, obtained from real-world clinical practice settings in > 11,000 patients, is the largest dataset to date of patients with brain cancer treated with TTFields. Moreover, it is one of the largest datasets of patients with GBM/high-grade glioma. TTFields treatment shows a favorable safety profile with localized, mild-to-moderate skin reactions that are often resoluble with over-the-counter topical ointments or by temporarily withholding TTFields treatment. Furthermore, no systemic effects or other treatment-related AEs were reported. As expected, the AE profile was comparable to published phase 3 TTFields/GBM clinical trial data and prior registry observations. Overall, the safety profile of TTFields remained consistent among patient subgroups (region, diagnosis, and age) and the total cohort, suggesting feasibility in multiple subpopulations, including elderly patients. These real-world global safety data confirm the known safety and tolerability of TTFields for GBM treatment.

## Data Availability

The datasets generated during and/or analyzed during the current study are available 3 years after date of publication.
