# Correction to: Global post‑marketing safety surveillance of Tumor Treating Fields (TTFields) in patients with high‑grade glioma in clinical practice

**DOI:** 10.1007/s11060-020-03681-8

**Published:** 2021-01-04

**Authors:** Wenyin Shi, Deborah T. Blumenthal, Nancy Ann Oberheim Bush, Sied Kebir, Rimas V. Lukas, Yoshihiro Muragaki, Jay‑Jiguang Zhu, Martin Glas

**Affiliations:** 1 Department of Radiation Oncology, Thomas Jefferson University, Philadelphia, PA 19107 USA; 2 Neuro‑Oncology Unit, Division of Oncology, Tel Aviv Sourasky Medical Center and Sackler School of Medicine, Tel Aviv University, Tel Aviv, Israel; 3 Department of Neurological Surgery and Neurology, University of California San Francisco, San Francisco, CA USA; 4 Division of Clinical Neurooncology, Department of Neurology, University Hospital Essen, University Duisburg-Essen, Essen, Germany; 5 West German Cancer Center, German Cancer Consortium, Partner Site Essen, University Hospital Essen, University Duisburg-Essen, Essen, Germany; 6 Department of Neurology, Northwestern University, Chicago, IL USA; 7 Lou & Jean Malnati Brain Tumor Institute at the Lurie Comprehensive Cancer Center, Northwestern University, Chicago, USA; 8 Faculty of Advanced Techno‑Surgery, Institute of Advanced Biomedical Engineering and Science, Tokyo Women’s Medical University, Tokyo, Japan; 9 Department of Neurosurgery, Tokyo Women’s Medical University, Tokyo, Japan; 10 McGovern Medical School and Memorial Hermann Hospital at Texas Medical Center, University of Texas Health Science Center at Houston, Houston, TX USA

## Correction to: Journal of Neuro-Oncology (2020) 148:489–500 10.1007/s11060-020-03540-6

The graphic abstract was missing in the original online publication. The original article has been corrected.

